# Influenza Nucleoprotein Delivered with Aluminium Salts Protects Mice from an Influenza A Virus That Expresses an Altered Nucleoprotein Sequence

**DOI:** 10.1371/journal.pone.0061775

**Published:** 2013-04-16

**Authors:** Megan K. L. MacLeod, Alexandria David, Niyun Jin, Laura Noges, Jieru Wang, John W. Kappler, Philippa Marrack

**Affiliations:** 1 Howard Hughes Medical Institute and Integrated Department of Immunology, National Jewish Health, Denver, Colorado, United States of America; 2 Department of Medicine, National Jewish Health, Denver, Colorado, United States of America; 3 Program in Biomolecular Structure, University of Colorado Denver, School of Medicine, Aurora, Colorado, United States of America; 4 Department of Medicine, University of Colorado Denver, School of Medicine, Aurora, Colorado, United States of America; 5 Department of Biochemistry and Molecular Genetics, University of Colorado Denver, School of Medicine, Aurora, Colorado, United States of America; Johns Hopkins University - Bloomberg School of Public Health, United States of America

## Abstract

Influenza virus poses a difficult challenge for protective immunity. This virus is adept at altering its surface proteins, the proteins that are the targets of neutralizing antibody. Consequently, each year a new vaccine must be developed to combat the current recirculating strains. A universal influenza vaccine that primes specific memory cells that recognise conserved parts of the virus could prove to be effective against both annual influenza variants and newly emergent potentially pandemic strains. Such a vaccine will have to contain a safe and effective adjuvant that can be used in individuals of all ages. We examine protection from viral challenge in mice vaccinated with the nucleoprotein from the PR8 strain of influenza A, a protein that is highly conserved across viral subtypes. Vaccination with nucleoprotein delivered with a universally used and safe adjuvant, composed of insoluble aluminium salts, provides protection against viruses that either express the same or an altered version of nucleoprotein. This protection correlated with the presence of nucleoprotein specific CD8 T cells in the lungs of infected animals at early time points after infection. In contrast, immunization with NP delivered with alum and the detoxified LPS adjuvant, monophosphoryl lipid A, provided some protection to the homologous viral strain but no protection against infection by influenza expressing a variant nucleoprotein. Together, these data point towards a vaccine solution for all influenza A subtypes.

## Introduction

Each year influenza virus A causes significant mortality and morbidity. These global outbreaks occur because of mutations or exchanges of the genes coding for the viral surface proteins, hemagglutinin and neuraminidase, processes known as antigenic drift and shift respectively [1]. Consequently, antibodies induced by prior viral exposure or vaccination are less likely to recognise and neutralise new variants. Current influenza vaccines are re-designed annually based on the sequences of the viruses predicted to dominate in the following year [2].

The unpredictable nature of antigenic changes makes it difficult to forecast which viral subtypes are likely to be circulating, strengthening interest in universal influenza vaccines [1]–[3]. Appropriately designed vaccines could be used to induce cross reactive antibodies against conserved portions of the viral surface proteins [4]–[6]. Alternatively, a cross reactive vaccine could take advantage of internal, less variable, proteins. For example, nucleoprotein (NP) is 90% conserved across viral variants with HLA class I and II epitopes often conserved [7]–[10]. Indeed, the presence of cross reactive T cell immunity is associated with protection in humans [11]–[13] and animals studies in which NP is delivered either as a recombinant protein with various adjuvants, in DNA vaccines, or in recombinant viruses or bacteria, have demonstrated that NP specific immunity is protective [14]–[19].

Many studies of potential influenza vaccines have used adjuvants or delivery methods that have not been approved for widespread use in humans. We recently demonstrated that, in mice, CD8 T cells specific for the D^b^ NP immunodominant epitope, NP_366–74_, primed with the universally used adjuvant, aluminium salts (alum), provide some protection from infectious challenge with an influenza A virus that expressed the same NP sequence as the priming antigen [16]. Addition of monophosphoryl lipid A (MPL), a detoxified form of LPS, improved the protective capacity of the vaccine. This approach is of limited value in an outbred population in which specific NP epitopes will vary depending on the individual’s MHC type. This prompted us to determine whether similar, or even better, protection could be obtained following immunization with whole NP.

Here we report that immunization of mice with recombinant NP and alum provides protection to influenza A challenge with viruses that either express the same NP as the immunizing antigen or a variant. We have examined the immune response and level of protection in C57BL/6 mice as this allows us to assess the adaptive endogenous T cell response using MHC tetramers in addition to the evaluating the antibody response using a NP specific ELISA. Following immunization and subsequent challenge with influenza viruses that express the same or a distinct NP, protection was associated with the presence of NP specific CD8 T cells in the lungs. In contrast to our previous study, the addition of MPL to the vaccination did not increase protection. Rather, the addition of MPL inhibited the protection afforded by vaccination with NP and alum.

This current study offers evidence for a safe, effective universal influenza vaccine strategy that could greatly reduce the economic, medical and human costs of influenza infection.

## Materials and Methods

### Mice and Infections

All animal experiments carried out in this study were done so at National Jewish Health in strict accordance with the recommendations in the Guide for the Care and Use of Laboratory Animals of the National Institutes of Health. The protocol was approved by the National Jewish Health Institutional Animal Care and Use Committee (IACUC), protocol number: AS2787. Mice were maintained in a specific-pathogen-free environment and all efforts were made to minimize suffering.

Female C57BL/6 (B6) mice (The Jackson Laboratory) were age-matched within experiments and primed at 7–10 weeks of age with 10 µg of NP protein delivered with 100 µg of alum (Alhydrogel, Brenntag) with or without 10 µg of MPL (Invivogen). Proteins were tumbled with alum with/out MPL for 2 hours at room temperature prior to injection. Mice were immunized intramuscularly (i.m.) in both hind-legs. Infections with A/Puerto Rico/8/34 (PR8) or pandemic H1N1 influenza A virus, A/New York/1682/2009 (NY1682) [20] were carried out on mice anesthetized with isofluorane and 50 µl of PBS containing 3PFU of PR8 or 4PFU NY1682-WT virus injected i.n. These doses were used as previous experiments showed that they led to approximately 20% weight loss in infected naïve animals. NY1682-WT was supplied by Dr. David Wentworth (J.Craig Venter Institute, USA). The virus was prepared following infection of confluent MDCK cells with virus stock for one hour. After four days of incubation, supernatant containing the virus was harvested and cell debris removed. Viral stocks were aliquoted and stored at −80°C.

### NP Protein

The NP sequence of PR8 was cloned from a pcDNA construct from Dr. Paul Digard (Cambridge University, UK) into a baculovirus expression vector. A His tag was cloned into the C-terminus. Hi5 cells were infected with the NP-encoding baculovirus and cultured for three days at 27°C. The infected cells were collected, lysed and the supernatant treated with DNase and RNase. The protein was purified on a nickel column and endotoxin removed by treatment with triton X which was then removed using BioBeads (BioRad). The NP contained ≤3.5EU or 0.007 ng of LPS per injection, determined by the limulus amebocyte lysate test (Lonza). The purified protein was run on a protein gel that was stained with coomassie blue to confirm purity (Figure S1).

### Flow Cytometry

Lymph nodes and spleens were prepared at the indicated times and red blood cells lysed. One lung lobe taken from perfused mice was cut into small pieces and treated with collagenase and DNase (both from Sigma-Aldrich). Single cell suspensions were stained with MHC tetramers at 37°C for 2 hours. APC-D^b^/NP_366–74_ and PE-D^b^/PA_224–38_ were produced as described [21], PE-IA^b^/NP_311–25_ tetramer was provided by the NIH Tetramer Core Facility. Antibodies to surface proteins were added and the cells incubated for a further 20 minutes at 4°C. The antibodies used were: anti-CD4 or anti-CD8 APC-eFluor780, anti-B220, anti-F4/80, anti-CD4 eFluor450, anti-CD44 PerCP-Cy5.5 all from eBioscience, and anti-CD8 pacific blue, anti-CD27 FITC, anti-CD127 PE, anti-CCR7-APC, and anti-CCR7 biotin and also streptavidin Pe-Cy7 from BD.

In all cases, 2–5 million events were collected on a CyAn ADP (Dakocytomation), and data analyzed using FlowJo version 8 or 9 (Treestar). Tetramer+ cells were defined by gating on live (based on forward-side scatter characteristics), CD8 or CD4 cells that were negative for the following antigens (the dump gate): B220, F4/80, MHC class II and either CD4 or CD8 depending on whether the cells expected to bind the tetramer were MHCI or MHCII restricted.

### ELISA for NP Specific Antibodies

Serum was prepared from venous blood collected by cardiac puncture and seperated by centrifugation following clotting. The serum was frozen at −20C before analysis (within one month of collection). 96-well immulon plates (Thermo) were coated with the recombinant NP protein at 10 µg/ml in PBS. The plates were blocked with 10%FCS/PBS before serum samples were added and titrated. To determine relative units, we titrated, on each plate, serum from B6 mice containing NP specific IgG1 and IgG2c. The samples were incubated overnight at 4°C. Plates were washed and alkaline phosphatase conjugated anti-IgG1 or anti-IgG2a detection antibodies (Becton Dickinson) added. After the addition of p-nitrophenyl phosphate substrate, 405 nm absorbance was detected using an Elx808 microplate reader.

### Plaque Assay for Viral Titres

One lung lobe from mice infected 4–5 days previously was homogenized and supernatants frozen until use. A final concentration of 2 µg/ml TPCK trypsin was added to the diluted supernatant which was plated on confluent MDCK cells. The cells were incubated for 1 hour at 37°C, the supernatants removed, then 1% SeaKem agar (Lonza) containing media added and plates returned to 37°C. 72 hours later, agar containing neutral red was added and plaques counted after a further 24–36 hours of incubation.

### Statistics

To examine protection from influenza A challenge, the percentages of original weight on each day after infection in each experimental group was compared to that in control infected mice. Data are presented as indicated in the figure legends. The statistical significances were determined using Student’s two-tailed T test (when two groups are compared) or ANOVA (when more than two groups are compared) with GraphPad Prism software version 4.

## Results

### NP Protein Delivered with Alum Primes NP Specific T Cells and B Cells

**Figure 1:**
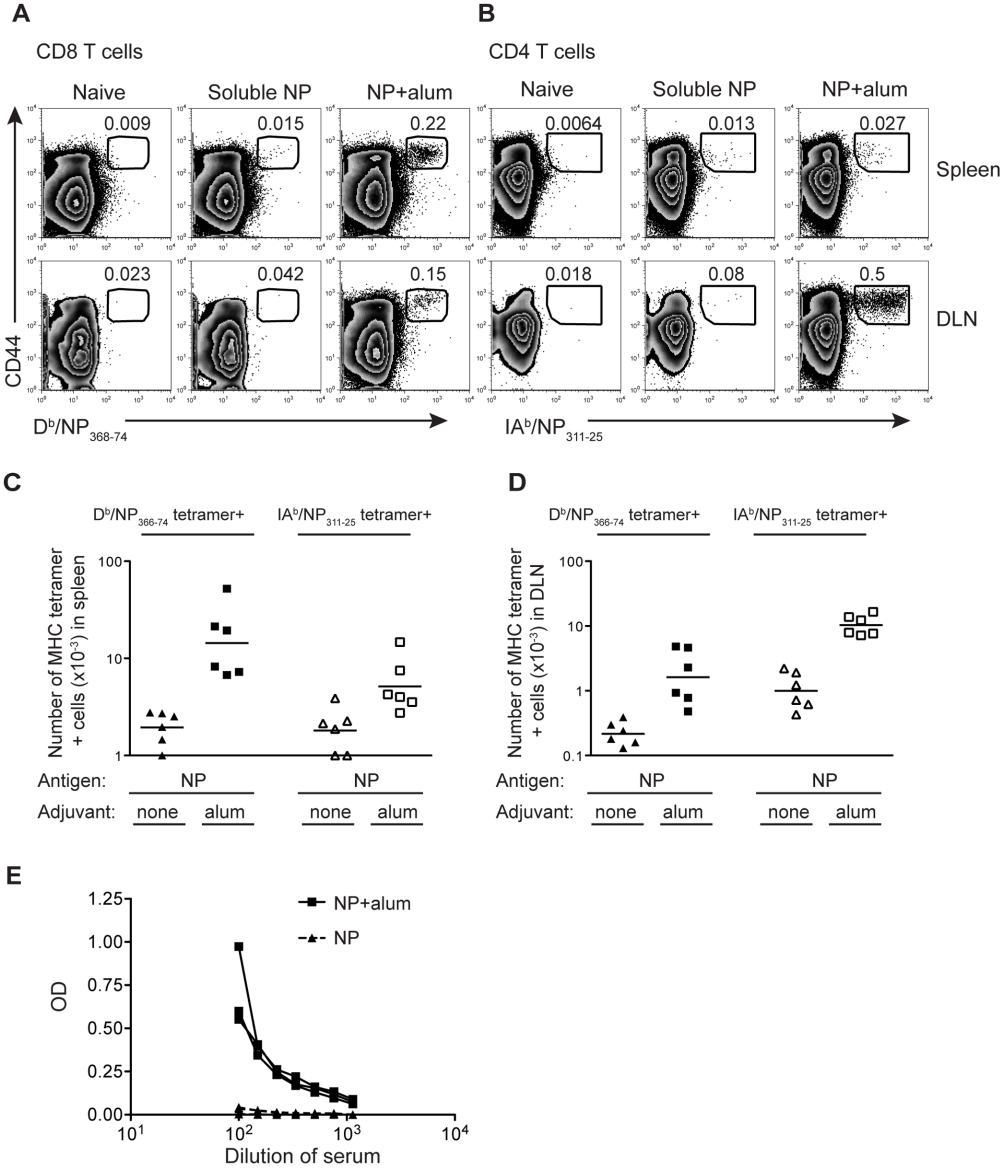
NP protein delivered with alum primes a specific T and B cell immune response. B6 mice were immunized i.m. in both hind-legs with a total of 10 µg of NP protein delivered with or without 100 µg of alum. The percentages (A, B) or numbers (C, D) of D^b^/NP_366–74_ CD8 or IA^b^/NP_311–25_ CD4 T cells were examined in the spleen or the two popliteal lymph nodes (DLN) 9 days later. Cells are gated on CD8+dump negative live cells (A) or CD4+dump negative live cells (B) with the number in the plot indicating the percentage of CD8 or CD4 cells that are CD44^hi^ tetramer+ as indicated by the gate. In C and D each point represents a mouse and the line shows the mean of the group. The X axis is set at the level of detection, determined by staining cells from naïve animals with the MHC tetramers. Serum from these animals was used to examine the level of NP specific IgG1 antibody (E), with each line representing one animal. These data are combined from 2 independent experiments with 3 mice per group.

We produced recombinant A/Puerto Rico/8/34 (PR8) NP protein in insect cells as this is a cost-effective method of producing recombinant protein that can easily be scaled up. Indeed, several bavulovirus based vaccines for human or veterinary use are available [22]. To demonstrate that our recombinant NP protein could prime an adaptive immune response, we compared immunization either with NP protein alone or delivered with alum following an intramuscular injection. The T cell specific response was measured by flow cytometry nine days post-immunization using MHC tetramers that bound either CD8 T cells specific for the D^b^ epitope, NP_366–74_, or CD4 T cells specific for the IA^b^ epitope, NP_311–25_, (Figure 1 A, B). Antigen specific T cells were detected in the spleen and draining lymph nodes (popliteal lymph nodes). The numbers of NP specific CD8 T cells were higher in the spleen than in the draining lymph nodes, whereas the reverse was true for NP-specific CD4 T cells. (Figure 1C, D).

While delivery of NP with alum clearly increased the expansion of antigen specific CD4 and CD8 T cells, immunization with the protein alone primed a small but detectable response (background set by staining cells from naïve age-matched mice with the MHC tetramers). This occurred despite prior treatment to remove contaminating nucleic acids and endotoxin (see Materials and Methods). In contrast, NP specific IgG, measured using an NP specific ELISA, was only present following immunization in the presence of adjuvant (Figure 1E).

### Immunization with Alum and NP Provides Complete Protection Against Challenge with an Influenza A Virus that Expresses NP with the Same Sequence as the Priming NP

**Figure 2:**
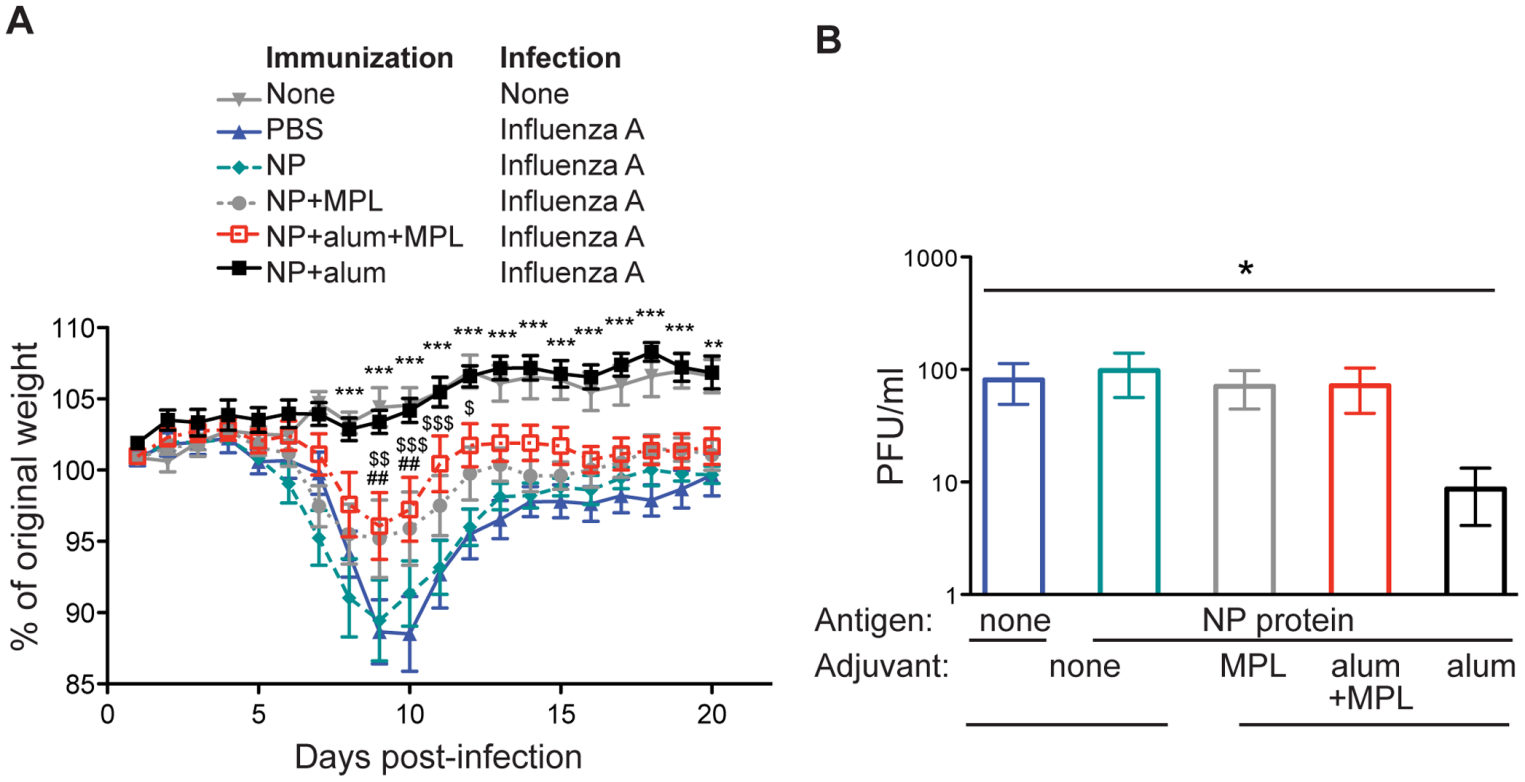
NP protein delivered with alum provides optimal protection from influenza A infection. B6 mice were immunized i.m. in both hind-legs with PBS (closed triangles) or a total of 10 µg of NP protein delivered alone (closed diamonds), or with 10 µg of MPL (closed circles), 100 µg alum (closed squares), or both adjuvants (open squares). At least 70 days later, these animals were infected with PR8 influenza A i.n. Mice were weighed daily and the percent of original weight calculated (A) or the amount of virus present in one lung lobe examined 4–5 days after infection (B). Data are combined from two separate experiments with 4–5 mice per group. In A, significant differences between the PBS control mice infected with PR8 and the experimental groups are indicated by the following symbol on the indicated days post-infection. Significant differences in mice immunized with NP and alum are indicated by **(p<0.01) and ***(p<0.001). Significant difference in mice immunized with NP and MPL are indicated by ## (p<0.01). Significant differences in mice immunized with NP and alum and MPL are indicated by $ (p<0.05), $$ (p<0.01), $$$ (p<0.001). No significant difference between control PBS mice and those immunized with NP protein were found. In B, * = p<0.05.

To examine protection afforded by immunization with NP protein, we infected mice that had been immunized at least 70 days earlier with NP protein administered either alone or with alum, MPL or both adjuvants (Figure 2A). These adjuvants were chosen as they are approved for use in human vaccines [23]. While the MPL used here is a generic form that may not act in exactly the same way as that present in licensed vaccines [24], the alum used in these studies is used in human vaccines [25]. In all the experiments here, the recombinant NP protein used was from the PR8 virus. Based on our previous findings using NP_366–74_ conjugated to an irrelevant protein, we expected that mice primed with whole NP and a combination of alum and MPL would be protected to the greatest extent [16]. However, while this group lost less weight than naïve animals, mice immunized with NP delivered with alum lost little to no weight. Immunization with NP protein and MPL provided a level of protection similar to that generated by immunization with the combination of adjuvants. The absence of weight loss in the NP and alum immunized mice was reflected in reduced levels of virus in these animals (Figure 2B).

### NP Specific CD8 T Cells in the Lung and Draining Lymph Node Early After Challenge Correlate with Protection

**Figure 3:**
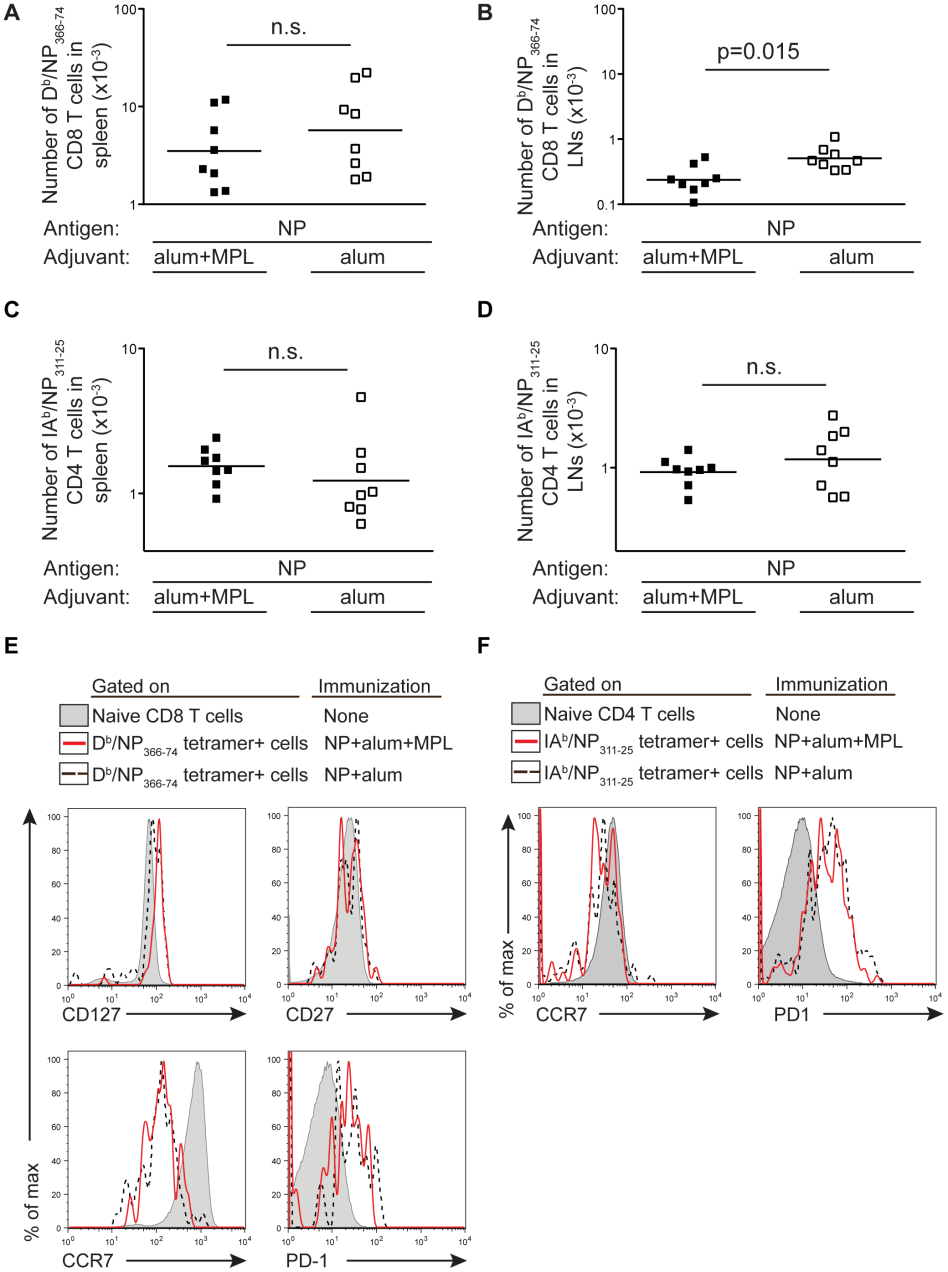
The NP specific memory T cell response is similar regardless of the adjuvant combination used. The numbers of memory NP specific memory CD8 (A, B) or CD4 (C, D) T cells were determined in the spleens (A, C) and popliteal lymph nodes (B, D) of B6 mice immunized with NP protein and alum and MPL or alum at least 70 days previously. Cells were gated as in Figure 1. The data are combined from two independent experiments with 4 mice per group indicated by each point, the line shows the mean of the group. The X axis is set at the level of detection. n.s. not significant. The expression of memory markers on either NP specific CD8 (E) or CD4 (F) T cells was examined in the spleen (CD8 T cells) or popliteal lymph nodes (CD4 T cells) at least 70 days after immunization. Cells are gated on naive/CD44 low CD8 or CD4 T cells (filled histogram) or on D^b^/NP_366–74_ tetramer positive CD8 memory T cells or IA^b^/NP_311–25_ tetramer positive CD4 memory T cells from mice immunized with NP protein and alum (black dashed line) or alum and MPL (red line). Tetramer positive cells were gated on live CD8 or CD4 positive cells that were negative for B220, F4/80, MHC II and either CD4 or CD8 respectively. The data show representative plots from 1–2 experiments with 4 mice per group.

The surprising finding that protection was more effective in mice primed with NP delivered with alum rather than the combination of alum and MPL led us to investigate which differences in the NP specific response induced by these immunizations might be responsible for the enhanced protection. The majority of the NP specific memory CD8 T cells were found in the spleen regardless of whether the mice were immunized with NP delivered with alum or alum and MPL (Figure 3A). Although there were slightly more NP specific memory CD8 T cells in the lymph nodes (popliteal) of mice immunised with NP and alum compared to those also immunised with MPL (Figure 3B), the total numbers of NP specific memory CD8 T cells in these animals were equivalent. The numbers of NP specific memory CD4 T cells in lymph nodes and spleens were also similar regardless of whether NP was administered with alum alone or in combination with MPL (Figure 3C–D).

It was possible that MPL altered the phenotype of the memory T cells. However, we found that the memory cells were similar in terms of the survival molecule, CD127, the chemokine receptor CCR7, and the costimulatory molecules PD-1 and CD27 (Figure 3C, D). This suggests that the generation of memory T cells after immunization with the various adjuvants could not explain the difference in protection.

To determine whether differences in the recall response of the memory T cells generated by immunization with NP delivered with alum or alum and MPL could explain the different levels of protection, we also examined the NP specific T cell response five days following viral challenge. We chose to examine the immune response five days after infection as this is before we observe any weight loss in the infected animals and, from our previous studies, we knew that the primary adaptive immune response to NP was not consistently measurable at day 5 [16]. Therefore, any T cell or antibody response present at this time must have been primed by the prior vaccination.

**Figure 4:**
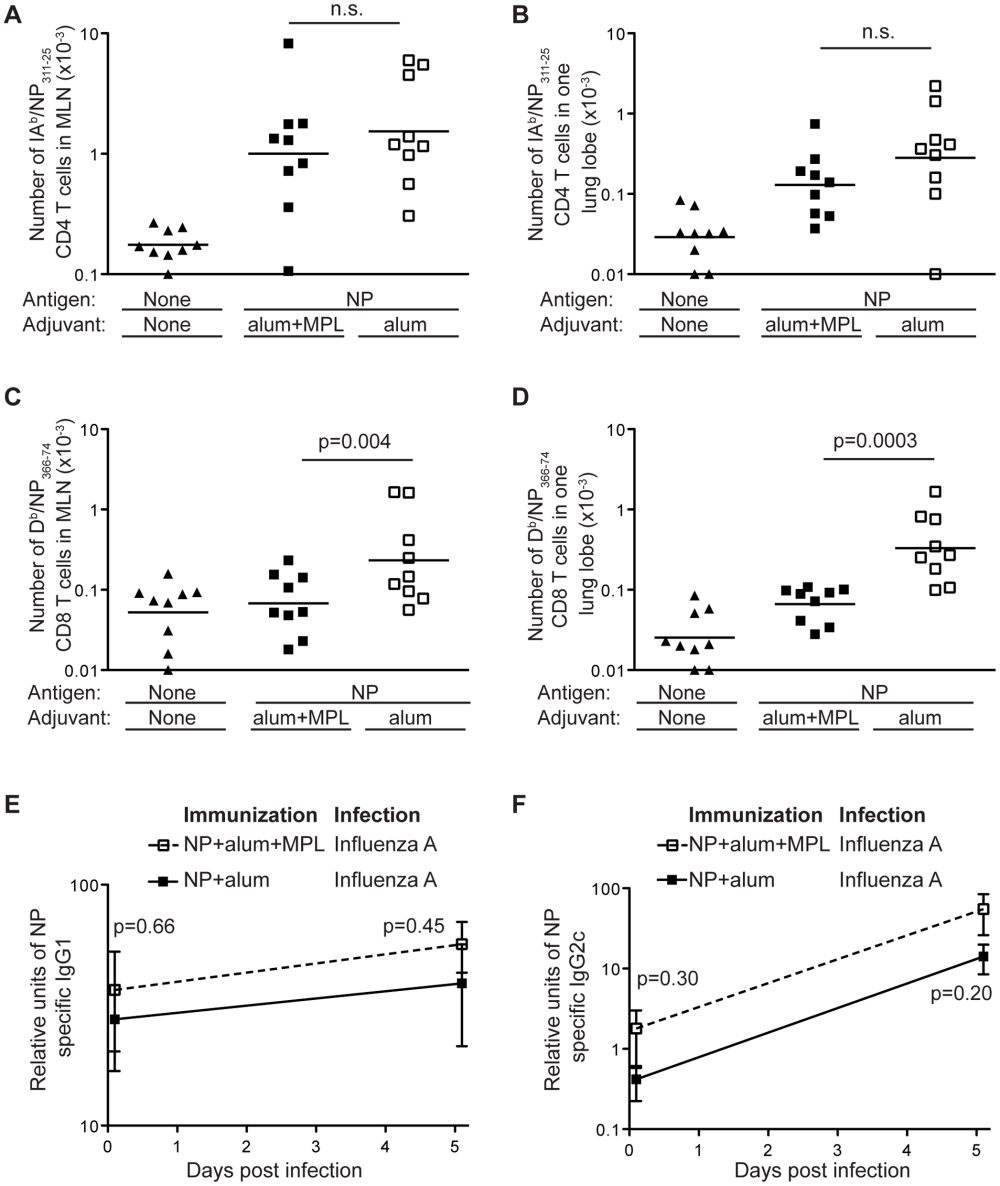
The number of D^b^/NP_366–74_ CD8 T cells is increased in protected animals. B6 mice were immunized i.m. in both hind-legs with PBS or a total of 10 µg of NP protein delivered with 100 µg alum with or without 10 µg of MPL. At least 70 days later these animals were infected with PR8 influenza A i.n. and the numbers of IA^b^/NP_311–25_ CD4+ T cells (A and B) or D^b^/NP_366–74_ CD8+ (C and D) were examined in the medistinal lymph node (A and C) or in one lung lobe (B and D) 5 days after infection. Cells were gated as in Figure 1. Each point represents a mouse and the line shows the mean of the group. The X axis is set at the level of detection. The relative amounts of NP specific IgG1 (E) and IgG2c (F) antibody in the serum of these animals were examined prior to and 5 days after infection, error bars show SEM. No NP specific antibodies could be detected at these time points in mice that had been infected but not immunized. Data are combined from two separate experiments with 4–5 mice per group. n.s.: not significant.

In the lung draining lymph nodes (the mediastinal lymph nodes) and the lung, NP specific CD4 T cells were detected in infected animals previously immunized with NP protein delivered with either alum or alum and MPL. No significant differences in this response were apparent between these two groups (Figures 4A and B), indicating that the enhanced protection observed in mice primed with alum was not a consequence of a NP specific CD4 T cell response. In contrast, the CD8 NP specific T cell response was greater in the mediastinal nodes and in lungs of mice primed with alum compared to those primed with the combined adjuvants (Figures 4C and D). This suggests that an enhanced NP specific CD8 T cell response in the lung and mediastinal nodes might be the cause of the increased protection.

NP specific antibody has been suggested to provide protection from influenza A infection in mice [26], [27]. We found that immunization with NP delivered either with alum or alum and MPL lead to the generation of similar levels of NP specific IgG1 and IgG2c (Figure 4E, F). Following infection, the levels of NP specific IgG2c increased steadily, and to a similar level, in both groups while only a modest increase in IgG1 was observed. The similar antibody response in these groups suggests that antibody is not responsible for the greater protection in mice immunized with NP and alum compared to those immunized with NP, alum and MPL.

### PR8 NP Protein Provides Cross Reactive Protection to Influenza A

**Figure 5:**
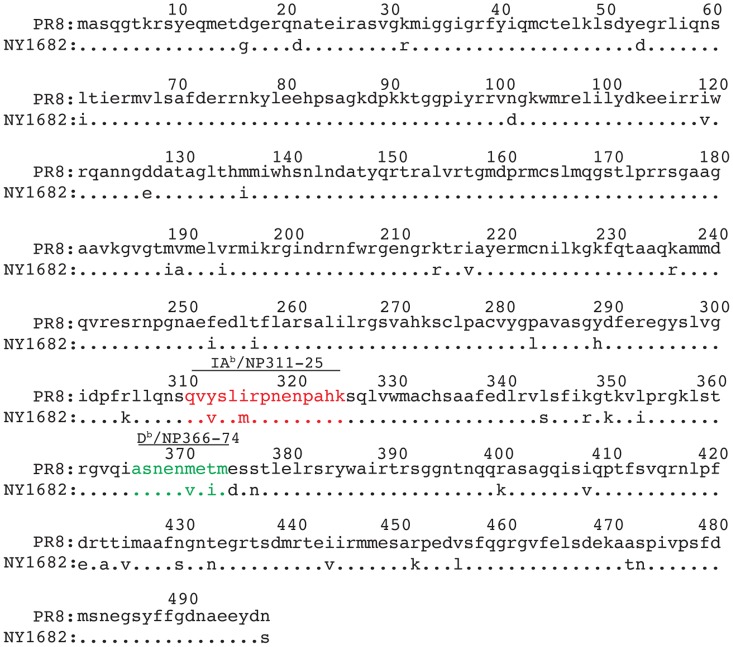
The NP amino acid sequence from PR8 and NY1682 influenza viruses differ in known epitopes. Sequence alignment for NP proteins from PR8 [46] and NY1682 [20]. The IA^b^ and D^b^ binding peptides are highlighted in red and green respectively.

**Figure 6:**
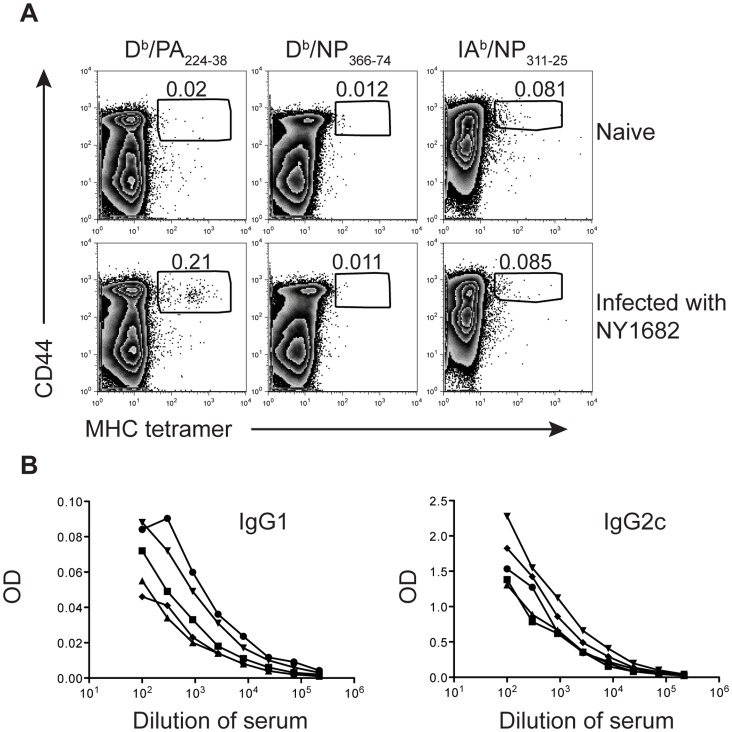
NY1682 infection does not prime T cells specific for immunodominant epitopes from PR8’s NP. B6 mice were infected (bottom) or not (top) with NY1682 i.n. and 25 days later the percentages of D^b^/PA_224–38_, D^b^/NP_366–74_ CD8 T cells, or IA^b^/NP_311–25_ CD4 in the MLN were examined (A). The numbers are the percentages of tetramer+CD44^hi^ cells out of gated CD8+ or CD4+ live cells that were also dump negative. The serum from these animals was tested for the presence of IgG1 and IgG2c antibody that bound to recombinant PR8 NP with each line representing one mouse (B).

While NP is highly conserved, variants do exist [9]. NY1682 was generated from a recombinant wild-type (WT) pandemic H1N1 influenza A virus, A/New York/1682/2009 (NY1682) [20]. The NP from this virus has 43 differences in amino acid sequence from that encoded by PR8. The differences include two changes each in the major peptides presented by D^b^ and IA^b^ to B6 CD8 and CD4 T cells respectively (Figure 5). Probably because of these differences, CD8 and CD4 T cells specific for PR8 NP peptides bound to D^b^ or IA^b^ could not be detected in the lymph nodes of NY1682 primary infected mice (Figure 6A). Cells from these animals did, however, bind to a D^b^/PA_224–38_ tetramer; PA_224–38_ is identical in PR8 and NY1682 [20], [28]. Moreover, cross reactive NP specific IgG1 and IgG2c antibodies, detected by ELISAs using recombinant PR8 NP, were clearly present in the serum of NY1682 infected animals (Figure 6B).

**Figure 7:**
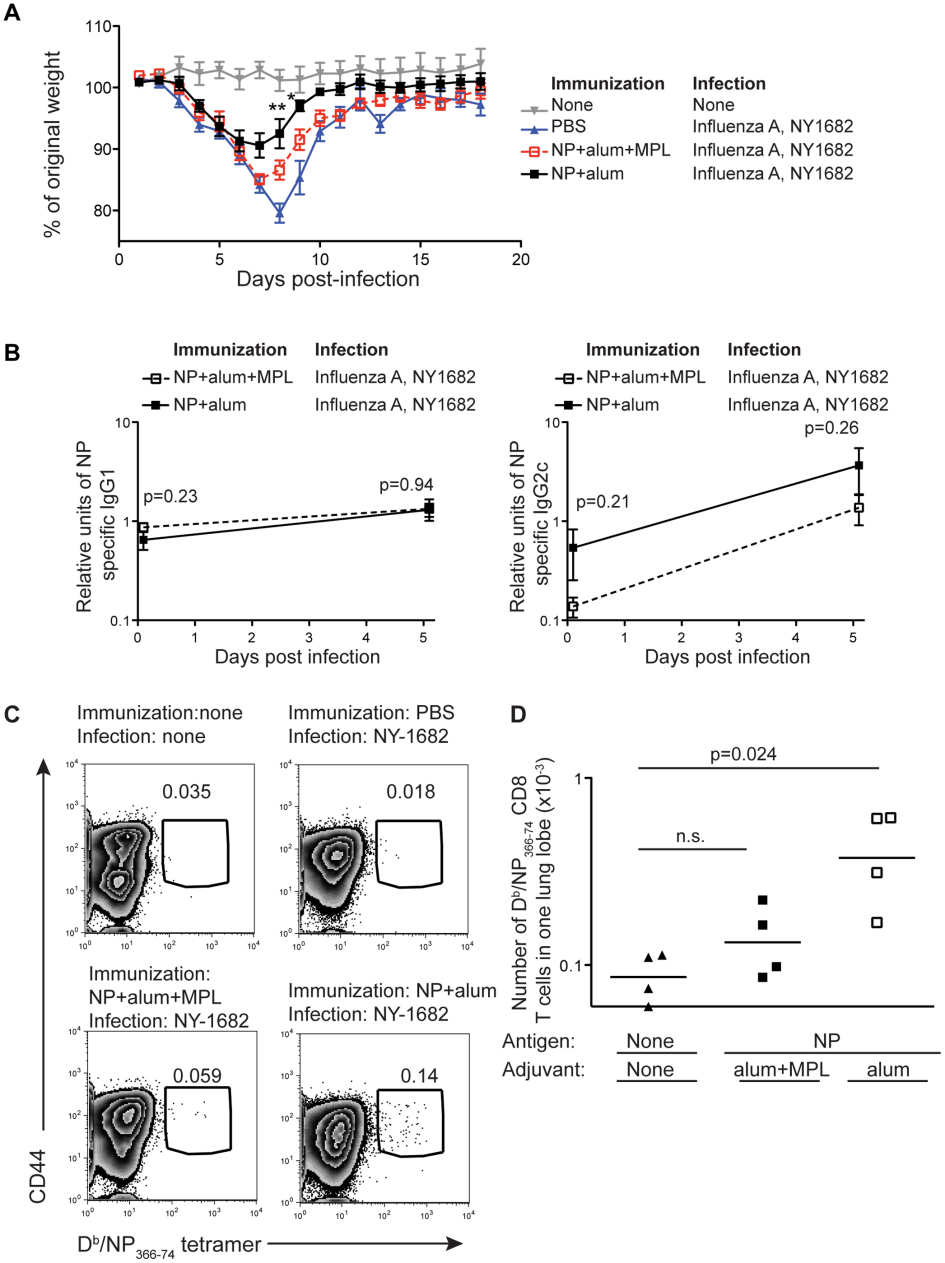
Immunization with PR8’s NP and alum primes protective immunity to NY1682. B6 mice were injected i.m. in both hind-legs with PBS (closed triangles) or a total of 10 µg of NP and 100 µg of alum with (open squares) or without (closed squares) 10 µg of MPL. At least 70 days later, these animals were infected with NY1682 i.n. The mice were weighed daily and the percent of original weight of each mouse calculated for each day. The data are combined from two experiments with 4–5 mice per group (A). These mice were bled one day prior to infection with NY1682 or 5 days following infection. The relative units of NP specific IgG1 and IgG2c present in the serum were determined using a NP specific ELISA (B). The percentages (C) or numbers (D) of D^b^/NP_366–74_ tetramer+ cells present in one lung lobe of these and naïve control animals were examined. In A, significant differences between the PBS control mice infected with NY1682 and those first immunized with PR8’s NP and alum are indicated with *(p<0.05) and **(p<0.01). No significant differences between PBS control mice and those first immunized with PR8’s NP and alum and MPL were found. In C, cells were gated on live CD8+ lymphocytes that were dump negative. Representative plots are shown from 1 experiment with 4 mice per group with numbers in the plot indicating the percentages of D^b^/NP_366–74_ tetramer+ out of gated live CD8+ cells. In D, each point represents a mouse and the line shows the mean of the group. The X-axis is set at the level of background staining.

To determine whether immunization with NP from one virus would protect from the morbidity caused by infection with a virus containing a different NP sequence, we immunized mice with NP from PR8. At least 70 days later these animals were infected with NY1682. Protection from infection with NY1682 was determined by weight loss (Figure 7A). Mice immunized with PR8’s NP delivered with alum and MPL lost about the same amount of weight as naïve animals. In contrast, while mice immunized with NP and alum did lose some weight, this was significantly less than that of the control group at the days of peak weight loss.

We tested whether the protection against weight loss afforded by vaccination with NP+alum correlated with any component of the NP specific adaptive immune response. Prior to infection, NP specific IgG1 and IgG2c responses were not significantly different between the groups of mice immunized with NP+ alum or NP+alum+MPL. Infection with NY1682 led to an increase in NP specific IgG2c but not IgG1 (Figure 7B) indicating that, at least, the IgG2c antibody recognised the NP from both PR8 and NY1682. However, these responses were the same in mice primed with NP delivered with alum or alum and MPL. Therefore, as only the animals primed with NP and alum showed any protective response, it seems unlikely that the NP specific antibody contributed to this protection.

Memory T cells can respond to altered versions of their epitopes [29], therefore, we examined whether we could detect NP specific cells with the IA^b^/NP_311–25_ and D^b^/NP_366–74_ tetramers containing the PR8 NP peptide sequences. No CD4 T cells that bound to the IA^b^/NP_311–25_ tetramer above background levels could be detected in either the mediastinal lymph node or the lung (data not shown). Reactivated D^b^/NP_366–74_ tetramer positive CD8 T cells were only detected in the lungs of mice primed with NP and alum then challenged with NY1682 (Figure 7C, D). Therefore, as following challenge with PR8, protection was associated with an early lung NP specific memory CD8 T cell response.

## Discussion

A universal influenza vaccine must protect against the virus regardless of viral subtype. By definition, the target must be well conserved and recognizable by an appropriate arm of the immune response. For this purpose, nucleoprotein fits the bill. There is evidence that NP might constitute a useful vaccine since, both in mice and humans, NP specific T cells can kill virally infected cells [12], [13], [30], [31]. We show that NP fully protects mice against pathology when the animals are challenged with virus expressing the immunizing NP sequence. The vaccine affords partial protection when mice are challenged with influenza expressing a different NP sequence. The NP of this challenge virus, NY1682, differs from that of PR8 at 43 out of 498 residues including two amino acid differences in each of the major peptides recognized by immunodominant CD4 and CD8 T cells. Despite this, we found that protection from the weight loss associated with influenza infection correlated with the presence of reactivated NP specific memory CD8 T cells in the lungs of the challenged mice.

To prime protective T cells by vaccination, some form of adjuvant is required. From a practical standpoint, a safe and widely used adjuvant offers the best approach. Here we have shown that delivery of NP to mice in the presence of alum primes at least some protective immunity against influenza. Importantly, alum delivered vaccines have been shown to prime IFNγ producing CD8 T cells in humans immunized with hepatitis B vaccine [32], [33]. Therefore, it is conceivable that an alum containing influenza vaccine would also prime CD8 T cells in humans.

While the live attenuated influenza vaccine is an attractive and feasible vaccine, the US CDC does not recommend its use in individuals under 2 or over 49 years of age, populations at substantial risk of complications following influenza infection [34]. Furthermore, the efficacy of this vaccine declines as early as a year following immunization perhaps because of the relatively short lifespan of mucosal immunity [35], [36]. A safe sub-unit vaccine capable of augmenting or, in individuals not exposed to influenza, priming, cross reactive immunity would be of tremendous value.

The trivalent influenza vaccine does contain some NP protein and can boost NP specific T cell responses [37]–[39]. In the US, however, this vaccine is given in the absence of an added adjuvant and is, therefore, unlikely to prime a protective cytotoxic CD8 T cell response. By adding alum and additional NP to the current vaccine, a multi-layered adaptive anti-influenza immune response will be induced offering a greater range of protection to the virus. Outside of the US, influenza vaccines containing either MF59 or ASO3 are available [40]. These adjuvanted vaccines can improve the anti-influenza antibody response to hemagglutinin and neuraminidase, whether they are capable of inducing protective influenza specific CD8 T cell responses is not known [41], [42].

We examined the NP specific adaptive immune response induced by immunization and following subsequent viral challenge. We used a mouse influenza infection model as this allowed us to determine the presence and phenotype of NP specific T cells following priming and following infection in the appropriate immune and peripheral organs. These experiments provide crucial information about which components of the adaptive immune response are involved in the protective response. However, mouse infections models do not always provide an accurate reflection of human immunity and these experiments would need to be validated in other influenza infection models.

The distinct protective response offered by immunization with NP delivered with alum in the absence or presence of MPL provided us with an opportunity to examine correlates of protection. The NP specific CD4 T cell and antibody responses were similar in animals immunized and challenged with the homologous PR8 virus regardless of whether MPL was present in the immunization. In addition, the antibody response following infection with NY1682, the influenza that expresses a distinct NP from PR8, was similar regardless of the adjuvants present in the immunization. Therefore, it seems unlikely that either the CD4 T cell or antibody responses could be responsible for the superior protection found in mice immunized with NP and alum alone.

In contrast, the presence of NP specific CD8 T cells in the lung draining lymph node and the lung itself correlated with reduced weight loss following infection with either an homologous virus or a virus that expressed an altered NP protein compared to that present in the vaccine. We tested protection following a dose of influenza that caused a sublethal infection in naïve mice. At higher doses of the virus the protection we observed may have been reduced. In our previous study, we also observed that protective immunity to influenza that expressed an homologous NP correlated with the presence of NP specific CD8 T cells in the lung at early timepoints after infection [16]. NP specific CD8 T cells in the lung that kill infected cells are known to provide protection from influenza, suggesting that in our studies, these cells provide protection via this mechanism [19], [43], [44].

The adjuvants that primed the best protection differed between our two studies. In our first study we primed only NP specific CD8 T cells. In this case a combination of alum and MPL induced the best protection. In the study described here, using intact NP and thus inducing NP-specific CD4 and CD8 T cells and antibody, alum alone induced the best protection. The reason for the difference is currently unclear. Following immunization with NP protein delivered with alum or alum and MPL, similar numbers of memory T cells and levels of antibody were generated suggesting that the generation of immune memory was similar regardless of the presence of MPL in the immunization. We speculate that differences in either the CD4 T cell or B cell responses in mice primed with both adjuvants somehow inhibited the ability of antigen presenting cells to reactivate the memory CD8 T cells [45].

We cannot rule out a defect in the generation of functional CD8 memory T cells in mice primed with both adjuvants although this seems unlikely given that CD8 T cells primed in the presence of both adjuvants in our previous study were not defective [16]. A change in the priming site may account for the difference as antigen and adjuvant was delivered i.p. in the previous study rather than i.m. in the studies presented here.

Regardless of the reasons for the superior protection following immunization with NP and alum compared to NP and both adjuvants, our immunization strategy offers a simple, safe and effective solution to the challenges of the variability of influenza virus. A universal influenza vaccine has the potential to offer significant protection to the world’s population and we show this is achievable using a safe, widely accepted adjuvant.

## Supporting Information

Figure S1 **The indicated ng of NP protein were run on a protein gel stained with coomassie blue.** Numbers on the right indicated molecular weight ladder (L) which was run in the lane on the furthest right.
(TIF)
