# Supplementary figures and images for: Influenza Nucleoprotein Delivered with Aluminium Salts Protects Mice from an Influenza A Virus That Expresses an Altered Nucleoprotein Sequence
Source: PLoS One. 2013 Apr 16;8(4):e61775. doi: 10.1371/journal.pone.0061775 (PMC3629017; doi:10.1371/journal.pone.0061775)

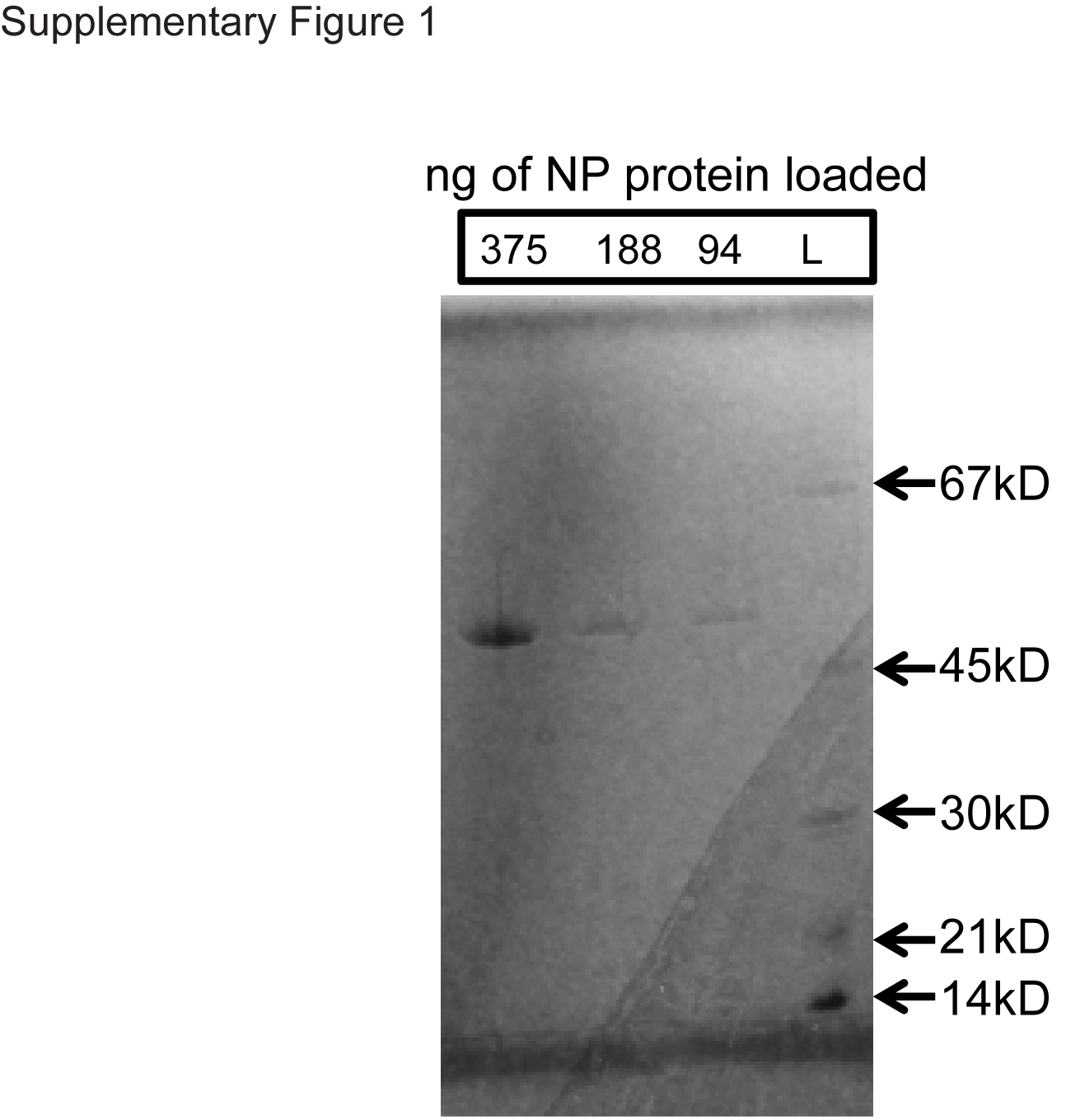

Supplement: Figure S1 — The indicated ng of NP protein were run on a protein gel stained with coomassie blue. Numbers on the right indicated molecular weight ladder (L) which was run in the lane on the furthest right. (TIF) [file pone.0061775.s001.tif]
